# Research on Control Strategy of Lower Limb Exoskeleton Robots: A Review

**DOI:** 10.3390/s26020355

**Published:** 2026-01-06

**Authors:** Xin Xu, Changbing Chen, Zuo Sun, Wenhao Xian, Long Ma, Yingjie Liu

**Affiliations:** China Coal Research Institute, Beijing 100013, China

**Keywords:** lower limb exoskeleton robots, control strategy, human–robot interaction, end-effector control, adaptive control

## Abstract

With an aging population and the high incidence of neurological diseases, rehabilitative lower limb exoskeleton robots, as a wearable assistance device, present important application prospects in gait training and human function recovery. As the core of human–computer interaction, control strategy directly determines the exoskeleton’s ability to perceive and respond to human movement intentions. This paper focuses on the control strategies of rehabilitative lower limb exoskeleton robots. Based on the typical hierarchical control architecture of “perception–decision–execution,” it systematically reviews recent research progress centered around four typical control tasks: trajectory reproduction, motion following, Assist-As-Needed (AAN), and motion intention prediction. It emphasizes analyzing the core mechanisms, applicable scenarios, and technical characteristics of different control strategies. Furthermore, from the perspectives of drive system and control coupling, multi-source perception, and the universality and individual adaptability of control algorithms, it summarizes the key challenges and common technical constraints currently faced by control strategies. This article innovatively separates the end-effector control strategy from the hardware implementation to provide support for a universal control framework for exoskeletons.

## 1. Introduction

With the intensifying trend of an aging population and the continuous rise in the incidence of neurological-related diseases, the number of people suffering from lower limb motor dysfunction caused by conditions such as stroke, spinal cord injury, cerebral palsy, and Parkinson’s disease is increasing annually. Globally, approximately 150 million individuals face varying degrees of motor dysfunction. These patients commonly experience issues including abnormal gait, insufficient muscle strength, and even paralysis, which severely impair their ability to perform daily activities and participate in society [1,2,3,4]. Traditional rehabilitation methods primarily rely on manual assistance from rehabilitation therapists to conduct training. Not only are these methods labor-intensive and poorly repeatable, but they also lack quantitative metrics, making it difficult to meet the current practical demands for efficient and personalized rehabilitation [5,6,7].

As a typical wearable intelligent device, the lower limb exoskeleton controls lower limb joints such as the hip, knee, and ankle via actuators to achieve coordination with the human movement process, thereby assisting patients in completing gait training [8,9]. From a functional perspective, lower limb exoskeletons can be categorized into two main types: rehabilitative and augmentative. Rehabilitative lower limb exoskeletons can be further divided into two subtypes: motion compensation type and gait correction type [10]. Each leg of the human lower limb typically has seven degrees of freedom (DOF). Exoskeleton robots often selectively control some or all of these DOFs based on task requirements. Through coordinated multi-joint movement, they can better simulate natural gait and enhance the wearer’s comfort and interactive compliance [11,12,13,14].

**Figure 1:**
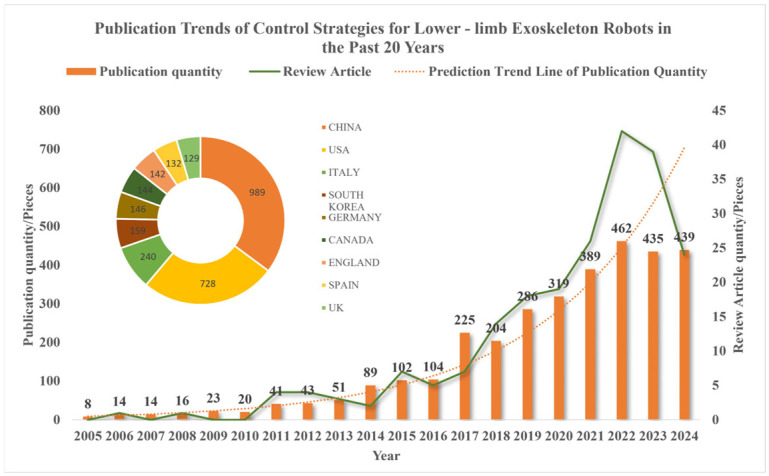
Statistical chart of the number of papers related to lower limb exoskeleton robot control.

The control system serves as the core of information interaction between the exoskeleton and the human body, undertaking the key roles of perceiving the user’s state, interpreting movement intentions, and implementing responsive control. In recent years, the number of research papers related to the control of lower limb exoskeleton robots has increased significantly, as shown in Figure 1.

This paper searches the web of science using the keywords “lower exoskeleton” and “control” for the number of published articles (the core search string is “lower exoskeleton” AND “control”, the search date is 28 May 2025, and the document types are review and article) and the proportion of countries in the past 20 years (2005–2024). The article’s inclusion criteria focus on the following: (1) journal articles and top conference proceedings; (2) research that proposes or verifies lower limb robot exoskeleton control algorithms. Exclusion criteria included the following: (1) studies focusing solely on mechanical structures; (2) upper limb exoskeletons or smart prosthetic devices. All related articles and review articles have shown an overall upward trend in the past 20 years, but have declined in the past two years. Especially with 2017 as the dividing line, the number of published articles has increased significantly.

When viewed on a five-year scale, the number of published articles has maintained a consistent upward trajectory. Among the contributing countries, China, the United States, and Italy are the major ones, accounting for over 50% of the total publications. This indicates that with the growing social emphasis on quality of life and rehabilitation products in recent years, the perception, decision-making, and control aspects of lower limb exoskeleton robots have gradually gained attention, and significant breakthroughs have been achieved in both relevant theoretical and practical research. Based on the statistics of the number of literature, this article extracts relevant research hot issues and difficult issues, and also evaluates the gap between the research content of this article and the published content, thereby providing an important perspective on the practical application of current exoskeleton control strategies.

Currently, a variety of commercial or experimental lower limb exoskeleton products have realized multi-degrees-of-freedom cooperative control, and are equipped with certain adaptive capabilities and biofeedback functions, which have significantly promoted the development of rehabilitation engineering and robot-assisted medical care [15,16]. A summary of some lower limb exoskeleton robot products is as shown in Table 1.

Although research on lower limb exoskeleton robots has shown an active trend in recent years, they still face three core challenges in practical applications:

Human Intention Detection: How to accurately recognize the user’s movement intention to achieve precise assistance.

Cooperative Motion Control: After acquiring the movement intention, how to achieve DOF and multi-joint coordinated control to assist the user’s movement naturally.

Parameter Optimization: How to dynamically optimize control parameters according to individual physiological differences.

**Figure 2:**
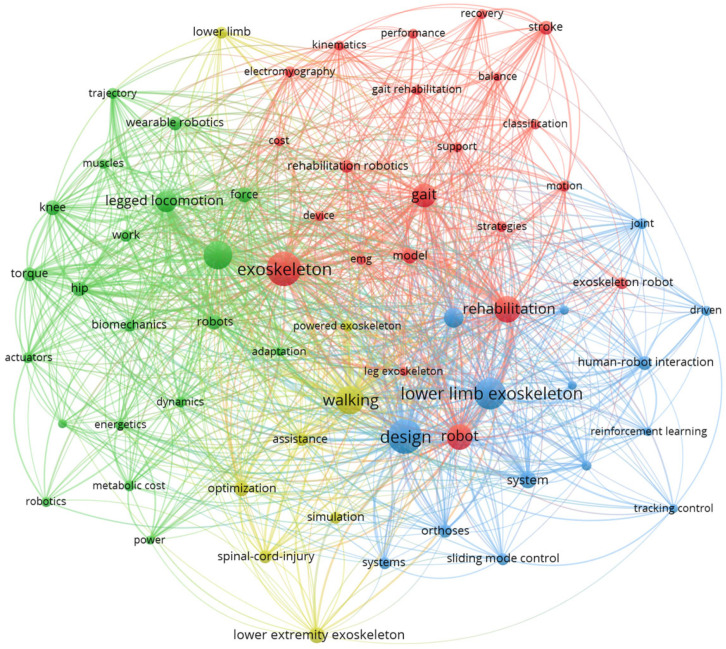
Visualization network analysis of keyword co-occurrence.

To deeply analyze the research hotspots and development context in the field of lower limb exoskeleton robots, a visual analysis of the literature was conducted on the Web of Science database using “lower limb exoskeleton” as the subject term, and a keyword co-occurrence visualization network was constructed, as shown in Figure 2. The results show that research in this field mainly focuses on four directions: first, rehabilitation and gait reconstruction (red cluster), which focuses on assisting patients with gait recovery through exoskeletons; second, biomechanics and motion analysis (green cluster), which emphasizes control methods based on dynamic modeling to improve the system and the natural movement of the human body in terms of coordination; third, control strategy and intelligent algorithm (blue cluster), which is the core direction of the current research, mainly exploring intelligent control methods such as human–machine collaboration, adaptive control, and deep reinforcement learning; fourth, system design and performance evaluation (yellow cluster), focusing on exoskeleton structure optimization, assistance effect, and energy consumption efficiency evaluation.

In summary, it can be seen that control strategy plays a core supporting role in all research directions and is the key to promoting the intelligence and practicality of lower limb exoskeleton robots. Its performance directly determines the naturalness and collaborative efficiency of human–computer interaction.

Traditional open-loop control or fixed-threshold feedback strategies often struggle to adapt to dynamic and variable human states [17]. In recent years, with the rapid development of high-resolution physiological sensing technology, human–robot dynamics modeling technology, and artificial intelligence methods, a series of advanced control strategies have gradually emerged, including proportional electromyography (EMG) control [18,19], variable gain control [20,21], AAN strategies [22,23], physiological model-based torque estimation [24,25], lower limb motion estimation based on physiological signals and muscle synergy [26], and artificial intelligence predictive control [27]. Although artificial intelligence algorithms have demonstrated superior performance in areas such as movement intention recognition, they have rarely been applied in end-control research, leading to a disconnect between the application of artificial intelligence in control and theoretical research [28,29,30].

**Figure 3:**
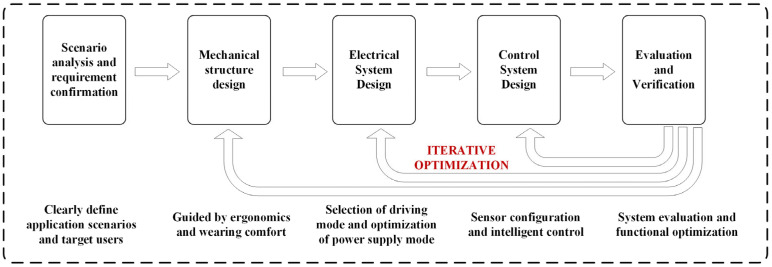
Design ideas and design process of lower limb exoskeleton robots.

The design of an exoskeleton control system depends not only on the target task but is also strongly influenced by the actuation mode, sensing structure, and human–robot interaction interface [31,32,33,34]. The design concept and design process of a lower limb exoskeleton robot are shown in Figure 3. Particularly in motor-driven systems, there is a high degree of coupling between control strategies and execution structures. The same control method is difficult to reuse across platforms, and it is challenging to elaborate on different actuation modes and their corresponding control strategies in a single article. Most review papers focus on hardware design or a single control method, lacking a synergistic analysis of actuation modes and control strategies.

To systematically review the current development of control strategies for rehabilitative lower limb exoskeletons, this paper uses control tasks as the main thread and categorizes them into four types based on clinical rehabilitation progress and human–robot interaction level. These categories are as follows: Trajectory Reproduction corresponds to the early stages of rehabilitation, and its goal is to prevent muscle atrophy through passive, robot-guided movement; Motion Following is an intermediate stage where the robot must follow user guidance to ensure comfort, focusing on motion synchronization; AAN represents the active recovery stage, where the robot dynamically adjusts assistance based on real-time performance to promote neural function remodeling; Motion Intention Prediction represents the advanced stage of daily assistance, where the control logic becomes actively perceptive, combining the horizontal coupling relationship of actuation–perception–execution, and taking “control tasks–control strategies” as the entry point. For different control tasks, it systematically reviews the research progress of lower limb exoskeleton control strategies, with a focus on the control implementation issues in motor-driven systems. The main contributions are as follows:

Horizontally compare the control system architectures and typical application scenarios under different actuation modes (motor drive, hydraulic drive, etc.), and vertically delve into various control strategies in the context of motor drive, systematically summarizing their mechanisms, advantages, disadvantages, and applicable scopes. By quantitatively comparing the tracking errors and response characteristics in recent studies, the trade-offs between control accuracy, system complexity, and human–machine interaction compliance were discussed.

Starting from the hierarchical control of exoskeleton robots, discuss the dependency relationships between control strategies, actuation modes, and sensing structures in combination with practical situations.

The previous research literature has mainly adopted a **hardware-centric approach**, classifying control strategies according to specific actuation types. This method often correlates control logic with actuators, and the performance of control strategies is liable to be masked by the physical limitations of the actuators. Unlike previous review papers, this article innovatively decouples end-effector control strategies from specific hardware implementations, providing a general framework that allows researchers to apply control theory to different types of actuators. It also focuses on the theoretical advancements and future development trends of end-effector control strategies. In addition to reviewing the existing literature, this paper elucidates the evolutionary trajectory of exoskeleton control—from fixed, non-interactive trajectory tracking control to intelligent, bio-signal-inspired, and human-intention-driven assistive control. By decoupling the control task from specific hardware, it also reveals how control strategies can be correlated with the patient’s motor function recovery stage.

## 2. Research on Drive Methods and Control System Frameworks for Lower Limb Exoskeleton Robots

**Figure 4:**
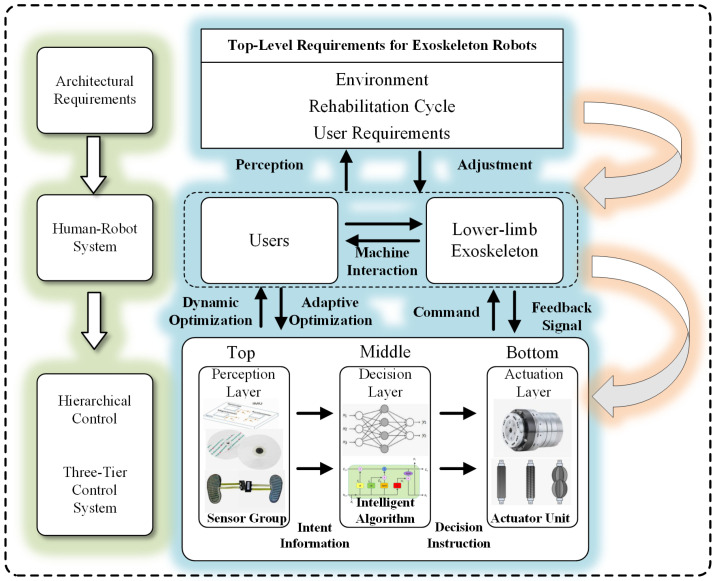
Overall control architecture of lower limb exoskeleton robots.

With the widespread application of lower limb exoskeleton robots in fields such as rehabilitation medicine and walking assistance, the design of their control systems has become crucial for achieving efficient human–robot collaboration. Lower limb exoskeletons are categorized into two main types: active control and passive control. Active control typically consists of three levels: perception, decision-making, and execution [35,36,37]. The overall control architecture of the lower limb exoskeleton robot is shown in Figure 4.

Perception Layer: Through multi-sensor fusion technology, physiological and motion information of the user is collected in real time, providing basic data for subsequent decision-making. For example, user information can be collected in real time through surface electromyography (sEMG) and IMU.

Decision-Making Layer: Using the collected data, combined with intelligent algorithms (such as machine learning, neural networks, etc.), the user’s movement intentions and states are analyzed, and corresponding control strategies are formulated. For example, the detected data can be analyzed to determine when the user transitions from walking on level ground to climbing stairs, planning the corresponding gait curve or controller input curve.

Control Execution Layer: According to the output of the decision-making layer, the drive system is controlled to execute corresponding actions, realizing assistance for the user’s movement or rehabilitation training. For example, the motor ensures precise output of the target torque or angle even under external disturbances.

Control strategies can be classified into three levels based on complexity and automation level:

(a) High-level control strategies: Can automatically detect terrain and user intentions based on multi-sensors to achieve autonomous control.
(b) Mid-level control strategies: Realize the analysis and execution of lower limb movements based on manual settings by the user or continuous state estimation.
(c) Low-level control strategies: Rely entirely on position or torque controllers to execute preset actions, with no ability to modify training trajectories.

The “perception–decision–execution” process is organically coupled. Among them, the physical carrier of the execution layer is the drive system, which directly affects the control effect of the exoskeleton robot. An ideal drive system should have characteristics such as high power density, good compliance, low power consumption, and compact structure. Currently, no single drive technology can meet all these requirements simultaneously. Table 2 systematically compares current typical drive modes across key dimensions, including technical implementation, control complexity, power output, structural compliance, system cost, and applicable scenarios.

Rotary motors and elastic actuators exhibit the best performance in terms of low cost, lightweight design, and reliability. Hydraulic and pneumatic systems, although capable of providing high driving force, are limited in their popularization by high costs, heavy weight, and high maintenance requirements; they are therefore suitable for high-intensity industrial applications. In contrast, pneumatic artificial muscles or cable-driven systems possess unique advantages in flexible and bionic robot design [57].

In summary, the high bandwidth and precise controllability of motor drives enable the implementation of almost all mainstream control strategies, which is crucial for lower limb exoskeletons in rehabilitation environments. In contrast, hydraulic, pneumatic muscle, and cable drives face significant challenges in implementing complex control strategies. Although these drive methods are beneficial for the compliance of AAN strategies, their highly nonlinear characteristics and time delays greatly increase the difficulty of implementing complex motion intention prediction control and hybrid force/position control. Therefore, motor drives have become the mainstream choice for current research and applications of lower limb exoskeletons. This paper will subsequently focus on motor drive systems and delve into their control tasks and strategy implementation paths.

## 3. Development Status of Control Strategies for Lower Limb Exoskeleton Robots

Section 2 analyzed the characteristics of different driving methods and clarified that the physical structure determines the overall performance of the exoskeleton robot system. To overcome hardware limitations and constraints and achieve more effective human–robot interaction, this section focuses on control strategies to evaluate how different control strategies adapt to or compensate for the characteristics of the physical structure.

In the application of lower limb exoskeletons, control tasks not only reflect the complexity of human–robot interaction but also directly determine the design depth of control strategies. To provide a structured analysis, this paper categorizes control tasks into four types based on patient treatment progress and the depth of human–machine interaction, as shown in Table 3. Although there is overlap between the boundaries of the four types of control tasks, such as follow-up control and AAN, the former aims to minimize interaction forces, while the latter purposefully adjusts assistance to provide only the necessary support. This classification method focuses more on the main clinical objectives of each task.

1. Human Motion Reproduction: This task mostly occurs in the early stage of medical rehabilitation, similar to the trajectory control of ordinary robots. It is relatively simple and does not require human feedback, only needing position or torque feedback.
2. Human Motion Following: This refers to ensuring that the exoskeleton’s movement is consistent with the human body. For the exoskeleton to achieve real-time following, human–robot interaction information is required as feedback—for example, using pressure sensors to detect the interaction force between the human body and the exoskeleton structure.
3. Joint Torque Assist-As-Needed: This type of control task is similar to the function of human muscles. The focus of the control strategy is to appropriately provide driving force to drive the movement of human joints.
4. Human Motion Perception-Prediction and Compliant Control: Suitable for high-level intelligent rehabilitation or daily assistance scenarios, this task requires real-time inference of the user’s intention and dynamic adjustment of output, with extremely high requirements for intelligence and compliance.

Correspondingly, the accomplishment of these control tasks also requires matching control strategies. Among them, the first three types of control tasks are mainly exoskeleton-dominated: the exoskeleton robot fully drives or supports human movement, and these are collectively referred to as passive control. The first type of control task is further called pure passive control because it requires no subjective participation from the human body. The second and third types of control tasks are called semi-passive control (human–machine collaborative passive control) because they require human movement parameters or provide additional assistance when the human body exerts active force.

In contrast, the last type of control task is human-dominated: the human body generates movement intentions, which are perceived and interpreted by the exoskeleton’s sensors; the exoskeleton then cooperates with the human body to complete the intended movement. This is also referred to as active control.

The following sections will build upon these four control tasks, systematically analyzing control strategies designed for specific tasks, examining the implementation principles of different control strategies, evaluating how they translate control tasks into robot execution logic, and subsequently analyzing the inherent advantages and limitations of each method.

### 3.1. Human Motion Reproduction

In the early stages of rehabilitation training, patients do not yet have the ability to actively move, so exoskeleton robots often use purely passive control strategies to drive patients to complete basic gait actions through preset trajectories. This type of control task takes “accurately reproducing human movement trajectories” as its core goal, emphasizing the strong constraint execution of the desired trajectory by the exoskeleton. The parameters pay more attention to high stability and high precision, but it will ignore the differences in the movement abilities of different patients. It is a “robot-led” control method. Although it is less interactive, it can provide initial support to patients throughout the process and help induce neural reconstruction through repetitive movements.

Before control, it is crucial to accurately generate limb motion trajectories. Typically, the main methods for obtaining limb motion trajectories are listed in Table 4.

In the traditional industrial control field, servo control-related methods are often used to make the system output approach the expected value infinitely. These methods are also applicable to the passive control of exoskeleton robots, such as PID control and computed torque control (CTC). Liang et al. [71] adopted the traditional dual-loop PID control method combined with gravity compensation. Without relying on a model-based controller, they achieved stable and precise control of a 4-DOF lower limb exoskeleton robot. Laboratory tests showed that the actual motion trajectory was almost consistent with the preset trajectory (sine curve), with an average angle error of less than 0.01 rad.

Similarly, the optimization of PID algorithms in the industrial field can also be applied to exoskeleton robot control, such as fuzzy control logic, genetic algorithm optimization, and particle swarm optimization (PSO) [72].

For external disturbances and model errors, fuzzy control logic is usually integrated to optimize the PID controller. Based on the idea of fuzzy reasoning, the P, I, and D parameters are dynamically adjusted according to the real-time error and error change rate to achieve adaptive control. The fuzzy rule base enhances robustness, enabling the system to adapt to uncertain factors in exoskeletons and human–robot interaction, such as load changes and nonlinear friction. The dynamically adjustable parameters allow handling nonlinearity and large disturbances.

To address the limited adaptability of exoskeleton robots, Cardona et al. [73] innovatively integrated the dynamic and kinematic data of both the robot and the patient into the PD closed-loop control loop. By real-time acquisition of the patient’s motion kinematics and dynamics data, the gain values were automatically adjusted to achieve multi-functional personalized assistance. This approach considers the conditions of different patients while ensuring stable output. Although the adaptive PD controller can meet the requirements of experimental tests, the system performance can be further improved through sliding mode control (SMC) or robust control in subsequent studies.

In recent years, the use of meta-heuristic algorithms to optimize exoskeleton controllers has attracted increasing interest from academia and researchers. Meta-heuristic algorithms include single-based meta-heuristic algorithms and population-based meta-heuristic algorithms. For parameter optimization of complex, nonlinear control models such as lower limb exoskeleton robots, the latter is usually selected. Common population-based meta-heuristic methods include evolution-based algorithms (genetic algorithm (GA), differential evolution (DE)), swarm intelligence-based algorithms (particle swarm optimization (PSO)), whale optimization algorithm (WOA), and grasshopper optimization algorithm (GOA), etc. Liu et al. [74] combined the search and optimization capabilities of meta-heuristic algorithms with the closed-loop feedback mechanism of PID control, proposing an online adaptive PID controller. They modified the PSO algorithm based on a “leaving and re-searching” mechanism to avoid falling into local optima: the algorithm continuously tests whether the currently converged solution remains optimal; if not, the converged particles are made to leave their current positions and re-search locally to obtain a better solution, ultimately reducing the objective function value. Simulation tests on a 9-DOF exoskeleton robot showed that the exoskeleton could adjust PID gains in real time when disturbed, significantly reducing tracking errors and suppressing chattering. However, the suppression performance still depends on the stability of the exoskeleton itself.

Amiri et al. [75] proposed a method using genetic algorithms and PSO to tune the PID controller of a 4-DOF lower limb exoskeleton. The optimization algorithms automatically searched for the optimal combination of controller parameters to minimize errors, and through global search capabilities, they optimized the adaptability of parameters to system uncertainties. However, only simulations were conducted, without considering the impact of mechanical actuator noise and disturbances to patients. PSO is often used for PID controller parameter optimization due to it having a simple algorithm, few parameters to adjust, and high search efficiency, but it faces difficulties in parameter initialization. Building on this, the research team combined adaptive algorithms with the Ziegler–Nichols (Z-N) method, proposing adaptive particle swarm optimization (APSO) to tune controller gains [76]. The Z-N method was used for parameter initialization, and APSO was applied for further optimization. Simulation experiments on a 3-DOF exoskeleton robot showed that the coefficient of determination for all joints was greater than 99%, which could significantly reduce trajectory errors. However, this model is difficult to apply to different trajectories, requiring repeated parameter optimization and lacking true environmental “adaptability”.

Belkadi et al. [77] proposed a robust adaptive PID controller based on an improved PSO algorithm, aiming to find the optimal PID parameters. This algorithm was verified on a knee joint lower limb exoskeleton robot driven by a brushless DC motor. Compared with the traditional APSO algorithm, it reduced the chattering of control signals in trajectory tracking.

In addition, inspired by the PSO algorithm, Mirjalili et al. [78] proposed the whale optimization algorithm (WOA). By using a mathematical model to simulate the process of humpback whales searching for and catching prey, WOA is also a swarm intelligence optimization algorithm similar to PSO. It can be used for the parameter optimization iteration of PID controllers to improve controller accuracy and avoid falling into local optima. Moharam et al. [79] adjusted the PID controller based on hybrid differential evolution (DE) and PSO, which is more robust and efficient while maintaining fast convergence. Subsequently, inspired by the natural predation behavior of locusts, Saremi et al. proposed the grasshopper optimization algorithm (GOA). The position of a grasshopper is updated based on its current position, the global best position, and the positions of other grasshoppers in the swarm. This helps GOA avoid falling into local optima. Since then, many variants of the GOA have emerged, such as the binary GOA optimization algorithm [80], chaotic GOA [81], dynamic GOA [82], and fuzzy GOA [83], which focus on improving GOA. These optimization algorithms for PID controllers also provide references for the passive control of exoskeleton robots.

This pure passive rehabilitation training strategy is usually used in the early stage of rehabilitation. It does not require active force from the human body and only relies on predefined trajectories to drive the movement of human joints, preventing muscle necrosis. However, the original intention of the exoskeleton robot design is to take the human as the main body, i.e., “Man in Loop”. A human–robot coupled system is formed through the actual interaction between the human and the exoskeleton. Therefore, it is of great significance to fully consider the human in the control strategy.

### 3.2. Human Motion Following

This control task aims to achieve dynamic collaboration between the exoskeleton robot and the wearer, so that the robot’s movements can be synchronized with the human’s natural movements in real time. When the patient has the intention of autonomous movement, the exoskeleton can respond quickly, greatly enhancing the patient’s subjective participation and autonomy in rehabilitation training movements. This control method not only improves the wearer’s comfort and interactive experience, but also promotes the functional reconstruction of the neuromuscular system.

The two core issues of follow-up control are improving tracking accuracy and reducing control delay. However, the exoskeleton will inevitably be affected by external disturbances during the following process, leading to challenges of modeling uncertainty, external disturbances, and time-varying dynamic parameters in the exoskeleton control process. Researchers have conducted extensive explorations from robust control, adaptive control, intelligent algorithms, and other aspects to enhance the dynamic response capability of the exoskeleton system in complex environments. Khamar et al. used a Nonlinear Disturbance Observer (NDO) to reduce the impact of uncertainty and external disturbances on the modeling of the entire system, and proposed a Backstepping Sliding Control (BSC) method combined with a nonlinear observer, which was applied to the knee joint exoskeleton. This method reduced the tracking error (less than 0.08 rad) caused by external disturbances and the time to eliminate disturbances, effectively reducing the impact of uncertainty and external disturbances [84].

Zhou et al. [85] designed a PD closed-loop control system for an exoskeleton driven by a servo motor, and conducted simulation tests on the control system based on the exoskeleton dynamics model. The average tracking error of the hip joint was approximately 1.5794°, and the average tracking error of the knee joint was approximately 2.1452°, verifying the effectiveness of PD control in low-complexity exoskeleton systems.

During the tracking control of the lower limb exoskeleton, the initial condition deviation, joint resistance, structural vibration, and environmental interference are all time-varying and uncertain, with unknown boundaries. Tian et al. [86] proposed an adaptive robust control method based on the Udwadia–Kalaba theory to solve the trajectory following problem of lower limb exoskeleton robots, expressing the trajectory tracking problem as a servo constraint problem. Without linearization or approximation, under the two degrees of freedom of the hip and knee joints, the trajectory tracking error quickly converges to zero, and the control torque is stable, which is significantly better than traditional methods. However, the parameters need to be tuned before use, and self-adaptation cannot be achieved.

To address the problem of poor motion control models for lower limb exoskeleton robots, Zhu et al. [87] proposed a lower limb assistive exoskeleton control method based on the Virtual Model Control (VMC) algorithm. The overall control goal was decomposed into a single-leg virtual model, the simplified Jacobian matrix was used to calculate joint torque, and model switching was performed in combination with gait rules and ground contact signals. This reduced the human–robot interaction force (the interaction forces in the pitch, horizontal, and vertical directions were reduced by 44.79%, 27.05%, and 56.04%, respectively), significantly improving the wearing experience.

In addition to the above-mentioned control optimization methods for model errors or disturbances, another way to solve nonlinear disturbances is to compensate for disturbances. With the development of machine learning and neural networks, neural networks have become another feasible tool for compensating for nonlinear disturbances or models. Asl et al. [88] used an adaptive feedforward neural network to compensate for the unknown nonlinear dynamics of the system. The neural network weight matrix only requires position information and not velocity information that is difficult to measure accurately. Tests were conducted on the three-degree-of-freedom exoskeleton robot TTI-Knuckle1, and the proposed controller’s tracking performance was comparable to existing methods, but with lower sensor measurement requirements.

To address this problem, Yang et al. [89] not only proposed a neural network to resist external disturbances but also considered the repeatability of system motion. Combined with Repetitive Learning Control (RLC), it learned periodic and non-periodic uncertainties, significantly improving the tracking accuracy of the lower limb exoskeleton robot. Tests were conducted on a two-degree-of-freedom exoskeleton robot, but no actual tests were performed.

In order to resist the influence of external interference and parameter uncertainty, fuzzy logic is often used to design controllers. Fuzzy controller is a control strategy based on fuzzy logic. By mapping input and output into fuzzy sets, fuzzy rules are used for reasoning and decision-making, and finally fuzzy output is generated, and the actual control output is obtained through the defuzzification process. This control method shows strong adaptability and robustness in the case of nonlinearity, complexity, and interference. Wu et al. [90] proposed an adaptive tracking control strategy based on the Fuzzy Cerebellar Model Articulation Controller (FCMAC) to address the control problem of exoskeletons under load changes, external disturbances, and other uncertainties. Computed Torque Control (CTC) was used for tracking, and FCMAC was used to resist model uncertainties and load changes. Accurate trajectory tracking could still be achieved under model parameter changes and load changes, and it had a high tolerance for uncertainties. Zhang et al. [91] proposed a Fuzzy Radial Basis Function Impedance (RBF-FVI) controller, which was applied to a six-degree-of-freedom lower limb rehabilitation exoskeleton robot. The controller adopted a dual-loop structure: the inner loop was a fuzzy position control loop, responsible for achieving precise control of training trajectory tracking and position compensation; the outer loop was a variable impedance control loop, which effectively compensated for nonlinear disturbances and modeling errors in the human–robot interaction process by dynamically adjusting the stiffness/damping parameters of the mechanical system. Experimental results showed that compared with the traditional PID control strategy, this scheme exhibited significant advantages in both gait trajectory following accuracy and system robustness.

Due to the nature of the sliding mode control, which uses a switching control law to make the system state switch at high frequency on both sides of the sliding surface to ensure that the system state moves along the sliding surface, delays in the switching process and external disturbances can cause errors in control. To correct the deviation, the control law will be further adjusted, causing high-frequency oscillations in the control input, and thus chattering occurs. In response to this, Liu et al. [92] designed a fuzzy adaptive sliding mode control system for a lower limb power-assisted exoskeleton. The dynamic model of the lower limb exoskeleton was established based on the Lagrange method, and in view of the difficulty in modeling the system nonlinearity and external disturbances, a fuzzy controller was introduced to adaptively adjust the sliding mode parameters. Simulations showed that this method improved the trajectory tracking accuracy while effectively reducing system chattering and enhancing the robustness and response speed of the control system.

In addition to the above optimizations for the controller, Li et al. [93] estimated the posture based on OpenPose-based visual feedback to achieve real-time and accurate following of the exoskeleton robot. An active human following algorithm was proposed to realize weight support and active human following. Zhang et al. [94] proposed a robust sliding mode adaptive control law to improve the following performance, and analyzed the tracking performance and adaptive characteristics of the lower limb joints based on the control law of a specific trajectory, resulting in strong following performance and anti-external interference ability.

Overall, the control strategies for following human motion are gradually evolving from passive tracking based on rigid trajectories to active perception and collaborative control, and the control goals are also expanding from single-joint tracking to multi-degree-of-freedom dynamic collaboration. Current mainstream research focuses on real-time dynamic collaborative control of multi-degree-of-freedom systems, the design of intelligent control algorithms with high robustness and strong adaptability, and the coupling optimization of exoskeletons and humans. However, in actual deployment, problems such as low modeling accuracy, perception lag, and strong dependence on controller parameters still exist, and there is an urgent need to promote the development of control algorithms toward self-learning, strong generalization, and cross-task transfer.

### 3.3. Joint Torque Assist-as-Needed

The high-precision trajectory tracking discussed earlier is effective for passive rehabilitation, but this method lacks active patient participation. To address the task of providing on-demand assistance for joint torque, the Assist-as-Needed (AAN) control strategy has gradually become a research hotspot. AAN is an intelligent control method that is centered on the user’s capabilities and dynamically adjusts the assistance force. It aims to provide “just the right amount” of torque support based on the wearer’s immediate state and functional needs.

This strategy breaks away from the limitations of full-time mandatory control, maximizes the user’s autonomous movement potential while ensuring safety, and features high personalization and adaptability. AAN control emphasizes dynamic collaboration between humans and machines and bidirectional information flow: when the patient has sufficient control capabilities, the system reduces intervention to allow them to complete movements actively; when muscle fatigue, gait imbalance, or weakened movement intention is detected, the system immediately intervenes to provide necessary assistance, thereby forming an active-passive integrated training mode. Significant progress has been made in research on AAN for lower limb exoskeleton robots [24,95], but traditional AAN requires complex sensing, prediction, and control methods, as well as precise mechanical structure linkage. In practical applications, AAN is often constrained by sensing burden and tuning dependence. Firstly, AAN relies on high-precision real-world data for feedback, and errors in this feedback are amplified by the algorithm and controller, severely impacting the human–computer interaction. Secondly, AAN has high demands on parameter sets; personalized parameters determine the effectiveness of personalized assistance. Future research should focus on adaptive algorithms and multi-sensor fusion to address these issues.

In addition to the AAN mode, for typical squatting movements, the squatting process does not require assistance from the lower limb exoskeleton robot, and only the standing-up process requires the exoskeleton robot to provide corresponding assistance. In this case, to simplify the exoskeleton structure, the electric actuator can be removed, and shape memory alloys can be used to achieve assistance without external energy input. Such exoskeleton robots are also called passive exoskeleton robots (unpowered exoskeleton robots). Its principle is mainly based on the combination of structural mechanics optimization and ergonomics; it does not require electrical energy and uses a lightweight support frame to directly transfer the load weight to the ground through a vertical transmission path, significantly reducing the load pressure on the human lower limb joints and muscle groups.

The main function of tendons is to transmit force between muscles and bones and they also play a role in energy storage. Therefore, the implementation of passive exoskeleton robots is generally as follows: a spring–damper composite device is integrated at the moving joints to simulate the characteristics of human tendons. When the wearer performs squatting movements, the elastic element absorbs kinetic energy through deformation and stores it as elastic potential energy, which is released at the appropriate stage of the movement cycle to assist limb movement, thereby reducing the need for active muscle force. This type of exoskeleton robot has low cost, lightweight, and a simple structure, and is suitable for simple scenarios that require continuous assistance, such as carrying and transporting on flat ground.

In 1890, Yagn published a “jumping walking” patent, which is known as the pioneer of passive lower limb exoskeletons [96]. Initially, a large spring was used to store energy; with improvements to the structure, a compressed airbag was finally used for energy storage. With the development of technology, current passive exoskeletons often use fluid springs, spiral springs, shape memory alloys, and elastic soft materials as core components to achieve functions including controlling mechanical movement, storing and releasing energy, and absorbing vibrations. However, current passive exoskeleton robots still have many limitations in applications with high movement intensity and complex terrain, including the following:

1. Conflict between assistive effect and human–machine interaction: Load capacity is a key performance indicator of the exoskeleton assist system. Compared with electrically driven exoskeletons, the effective load performance of passive exoskeleton systems still has a significant gap. The load-carrying capacity is directly related to the damping device, but excessive damping can easily cause discomfort to the user, and rigid materials can easily cause harm to the human body. Therefore, how to balance the assistive effect and human–machine interaction experience is one of the urgent problems to be solved for passive exoskeletons.
2. Need for further improvement in control accuracy: The biomechanical characteristics of the human movement system exhibit complex nonlinear features, and the relationship between muscle contraction degree and output force is complex. According to Hooke’s law, a single spring can only achieve linear assistance, which is contrary to the kinematic characteristics of the human body. Thus, control accuracy and assistive effect need to be improved.

In response to the above problems, many experts have made efforts. Based on the principle of human tendons, Wang et al. [97,98] designed a passive spring-driven ankle-foot exoskeleton robot to reduce human energy consumption by mechanically improving walking efficiency. An intelligent clutch without electronic sensors was proposed, which recognized the gait phase in real time through a rigid trigger mechanism and controlled the engagement and release of the spring to achieve precise passive assistance. Experiments showed that the activity of the soleus muscle was reduced by 72.2%, but the calf brace was prone to displacement, and there was still room for optimization in the spring stiffness range and material weight.

Although “intelligent clutch” technology has made important progress in passive exoskeletons, current designs still have shortcomings. First, the movement timing is fixed; the current clutch switching timing is determined by the position of the internal pin, which can only adapt to specific walking rhythms. Second, spring stiffness is single; the ordinary coil springs used currently can only provide fixed tightness and cannot adapt to different walking speeds and parameters. In response to this, Pardoe et al. [99] developed a passive ankle exoskeleton with an elastic pneumatic artificial muscle (PAM) as the energy storage element, weighing only 1.51 kg. At the same time, the clutch structure was improved and designed as a two-layer pawl that interacts with the ratchet gear and the engaging arm. PAM was used to simulate the nonlinear characteristics of human tendons, and precise assistance was achieved by combining an adjustable clutch. Tests were conducted on five participants, and the results showed that the exoskeleton could reduce the activation of the calf muscles; high-pressure PAM reduced the activation degree more than low-pressure PAM, but it would affect the range of motion. Therefore, it is necessary to balance the assistive performance and the range of motion to maximize the human–machine interaction effect. However, the matching degree between PAM stiffness and the user needs further optimization.

To reduce the weight of the exoskeleton robot itself, Leclair et al. [100] also used PAM to design a passive ankle exoskeleton. Unlike other existing devices, this passive exoskeleton can store a large amount of energy and release all the energy at once, accurately outputting a large torque during the push-off phase of the gait cycle.

Guan et al. [101,102] proposed a new passive energy-storing exoskeleton “ES-EXO” for patients with spinal cord injuries. The tension spring at the hip joint stores energy during the gait support phase and releases it during the swing phase to assist the patient in completing hip flexion and extension movements. By optimizing the energy storage element, the hip joint torque during walking was reduced, and experiments showed that the hip flexion torque was reduced by 37.2%.

The human joint is a complex and highly coupled system. Current passive exoskeleton robots pay less attention to the energy superposition effect between joints, the efficient utilization mechanism of gait energy, and the muscle synergy mechanism. Therefore, Wang et al. [103] proposed a passive lower limb exoskeleton robot with muscle force synergy compensation. Using the human lower limb dynamics model, the energy changes during walking were analyzed to obtain the mechanism and law of energy storage and release, simulating the energy cycle of muscles and tendons. The muscle force synergy compensation path for joint muscles was designed, and finally, elastic energy storage elements were designed based on the stiffness of the ankle and hip joints to store and release gait energy in a timely and appropriate amount, providing joint assistance along the muscle force compensation path to maximize the utilization efficiency of gait energy. Simulation analysis showed that the total metabolic energy consumption of the lower limb-related muscles within a single gait cycle was reduced by 15.5%. However, the elastic energy storage element generates vibration during the energy release process, which affects the assistive effect, and the spring stiffness remains unchanged, so the human–machine interaction effect needs to be improved.

During walking, there are significant differences in the force contribution of lower limb muscles; for users with gait problems, this difference is further amplified. The different layout positions of energy storage elements and clutches directly affect the assistive effect. However, passive exoskeletons themselves do not provide energy and are sensitive to their own weight, so their structure must be simplified. Therefore, designing an energy storage element and clutch with strong adaptability for different users is one of the urgent problems to be solved currently.

### 3.4. Perception–Prediction of Human Motion and Compliant Control

The exoskeleton systems mentioned above essentially do not consider the user’s active intentions. The original intention of the exoskeleton design is to influence the human body’s movement ability; if functions such as perception, decision-making, and adjustment are added, such exoskeleton robots will become intelligent agents capable of interacting with the human body and also become actively controllable exoskeleton robots. By increasing active participation, the patient’s dependence on robot assistance can be reduced by improving the effect of rehabilitation training. To achieve this effect, it is very important to integrate human consciousness or movement intentions into the control system, i.e., “Man in the Loop”. Without humans, signals cannot be perceived, and feedback signals and control signals for decision-making are lost, thus losing intelligent characteristics. Therefore, the human body itself is crucial in the structure of actively controlled exoskeleton robots.

The main difference between active exoskeletons and passive exoskeletons lies in their ability to judge the human body’s movement intentions in real time and modify control strategies in real time, fully considering human–machine interaction to achieve human–machine collaboration. This method mainly has two difficulties: (1) recognition and prediction of human motion intentions, and (2) control of the human–machine coupled dynamic system. There have been many studies on the former; motion intentions are recognized and predicted through sEMG signals, IMU signals, and human–machine contact pressure/torque signals. After correct and real-time prediction of human intentions, how to set the movement trajectory of the limbs and achieve compliant control effects is crucial. However, exoskeleton robots are strongly coupled and highly complex systems, making it difficult to obtain the most ideal control algorithms using classical control theories or modeling methods. At the same time, control strategies are strongly related to perceived signals, and there are currently few studies on universal control strategies. This section will conduct a detailed comparison of control strategies based on different perceived signals, focusing on the end-control part based on EMG and EEG, aiming to reveal the similar control ideas behind different control strategies.

Bioelectric signals can directly obtain the movement intentions of patients with lower limb movement disorders, which are ahead of the movements themselves and help to achieve more natural human–machine interaction. However, the methods for signal acquisition and signal processing are not yet mature and universal. Compared with physiological signals, physical signals have mature methods from perception to processing, but physical signals are only triggered after the human body produces actual movements, lagging behind human movements. Although data-driven methods are currently used to predict movements, combining LSTM or machine learning algorithms to predict future states using historical physical signal data, human movements have strong uncertainty, especially sudden movements. Therefore, using data-driven methods to establish a multi-modal database, combined with physiological signals, historical signals from physical sensors, and machine learning prediction algorithms, has become a solution to compensate for the lag problem of physical sensors [42,104].

EEG is the most commonly used brain signal in BCI applications. Compared with other brain electrical signal measurement methods, this method has the advantages of non-invasiveness and low cost, and only requires placing electrodes on the scalp for measurement. However, its signals are very weak (signal amplitude is at the microvolt level) and prone to artifacts.

**Figure 5:**
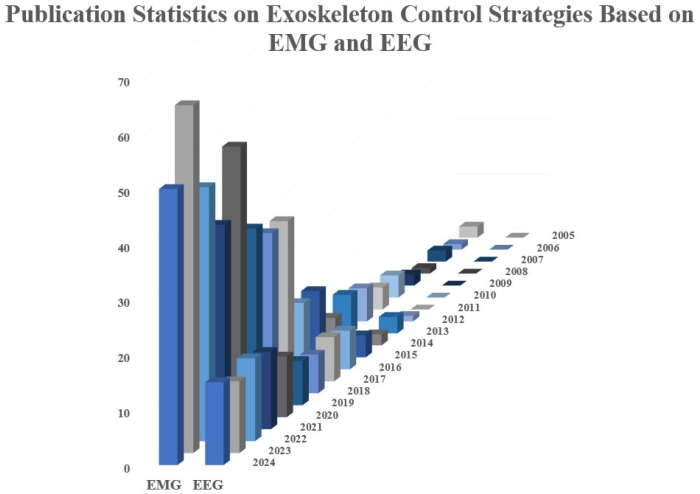
Comparison of studies with EMG and EEG as keywords.

In recent years, with the rapid development of sensing technology and artificial intelligence algorithms, the number of lower limb exoskeleton robot products with real-time perception and motion intention prediction capabilities has grown rapidly, and related research has also shown an explosive growth trend, as shown in Figure 5. In the Web of Science database, searching for published studies in the past 20 years (2005–2024) using keywords such as “lower limb exoskeleton”, “control”, “EMG”, or “EEG” shows that the number of EEG studies is only 18.7% of the number of EMG studies.

This phenomenon not only reflects the current mainstream research direction, but is also related to the differences in the complexity of acquisition and real-time processing of the two types of physiological signals. In contrast, EMG signals can obtain reliable motion information with a lower technical threshold, and are superior to EEG signals in terms of spatial resolution and response timeliness. Therefore, they have been more widely used and verified in motion intention decoding and power assist control.

#### 3.4.1. Based on sEMG Signals

sEMG signals are bioelectric currents generated by the contraction of surface muscles of the human body. The nervous system controls muscle activities (contraction or relaxation); at the same time, different muscle fiber motor units on the surface skin generate different signals, which can map the movement state of the human body. sEMG signals are non-stationary microelectric signals with an amplitude usually ranging from 0 to 1.5 mV. However, sEMG signals generally appear 30–150 ms earlier than limb movements, allowing for advance judgment of movements. Therefore, movements can be predicted through sEMG.

The proportional myoelectric method is an early researched and easy-to-implement method. Its main idea is to quantify the EMG signal, establish a mathematical relationship between it and the kinematics and dynamics of the human lower limbs, and set the input of the controller as the EMG signal, i.e., directly controlling the lower limb exoskeleton robot through the EMG signal, which is divided into fixed gain and variable gain.

Young et al. [105] compared the hip joint torque control strategy and the proportional myoelectric control strategy, verifying the effectiveness of this control strategy in reducing human metabolic consumption. However, the results were obtained using a single exoskeleton device with a specific control configuration when walking at a single speed level, and detailed data in complex terrains or for patients with motor function impairments cannot be tested. Ha et al. [106] proposed a volitional control method for providing powered knee joint assistance. Different from the above methods, instead of directly using EMG signals to control joint movements, the eigenvalues of surface EMG signals after quadratic discriminant analysis and principal component analysis were used in the impedance controller. Tests were conducted on three amputees, and the average root mean square trajectory tracking error was 6.2°.

Although the fixed gain controller is relatively simple in structure and implementation, its assist performance usually lacks flexibility and is difficult to dynamically adjust according to the user’s physiological needs in different exercise states. Therefore, when faced with complex scenarios such as changes in gait stages, load conditions, or user fatigue, its control performance and human–machine synergy will be greatly affected. In order to improve the adaptability and intelligence of the system, researchers have gradually turned their attention to variable gain proportional myoelectric control strategies to achieve more adaptable and flexible power assist control.

Koller et al. [107] proposed an adaptive gain proportional myoelectric control method for an ankle exoskeleton driven by pneumatic muscles, which mapped the myoelectric signal amplitude to the driving signal and dynamically adjusted the control gain according to real-time myoelectric characteristics. This method was experimentally verified in eight healthy subjects. By increasing the ankle joint assist force and reducing the hip joint force, metabolic consumption can be significantly reduced after a short period of training. This study not only verified the effectiveness of the variable gain control strategy in a dynamic environment, but also provided a general control idea for the motor-driven exoskeleton system, that is, a flexible control gain mapping relationship is constructed based on EMG signal changes, thereby achieving a more refined and flexible assist.

Tian et al. [108] proposed a gain-tuned compliance control strategy (EMG-based Gain-Tuned Compliance Control, EGCC) based on surface electromyography signals. By monitoring the sEMG signal in real time, normalizing it and mapping it to the gain parameter of the controller, the robot end-effector control effect can be dynamically adjusted. Experimental results in training modes such as continuous passive motion and straight leg raise show that the EGCC strategy can reduce human–machine contact force and improve training safety and comfort. However, the normalized threshold needs to be set manually, and the training mode is relatively single.

Grazi et al. [109] innovatively used the electromyographic signal from the medial head of the gastrocnemius muscle to control the flexion and extension movements of the hip joint. This method detects the envelope curve of the electromyographic signal during a specific phase of the gait cycle and multiplies it with a gain coefficient to generate assist torque. Experimental results show that this strategy not only avoids the discomfort and signal artifacts caused by placing electrodes at the human–robot interface, but also effectively reduces the muscle activity of the rectus femoris and tibialis anterior muscles, improving the synchronization of power assistance and user comfort.

Chen et al. (Document 24) used sEMG signals as the input of the exoskeleton perception module, used machine learning-based signal classification methods to identify motion states, controlled the exoskeleton robot to make corresponding movements through the upper computer, and fed back real-time data collected by position sensors to achieve closed-loop control and improve the accuracy of motion control. However, due to structural design limitations, there will be oscillating deviations between the actual trajectory and the ideal trajectory, and due to the control signals generated by the upper computer, the control real-time performance is poor.

As the patient’s rehabilitation progress improves, their dependence on the power assistance of the exoskeleton robot will become lower and lower. At the same time, to maximize the patient’s participation, the exoskeleton needs to provide the minimum possible assistance, i.e., the AAN mode. The principle of the AAN controller is that the exoskeleton predicts the subject’s autonomous input torque and then adjusts the motor output to complete the required trajectory. As a typical force–position hybrid control architecture, the AAN controller takes the prediction of human autonomous torque as a key input variable. By establishing a human–machine interaction dynamic model, it ensures trajectory tracking accuracy while accurately matching the actual assistance needs of the patient in the current rehabilitation stage. The torque prediction link, as the core technical support of the AAN controller, directly determines the dynamic adaptability of the assistance output and the efficiency of the human–machine collaboration. Therefore, many scholars directly estimate the joint torque of lower limb exoskeleton robots based on physiological signals.

Torque is divided into active torque and passive torque. Active torque is generated by muscle contraction and is responsible for completing movements; passive torque comes from skeletal muscles and ligaments and is responsible for stabilizing joints. Accurate prediction and output of active torque are crucial for the control of lower limb exoskeleton robots. Torque prediction usually adopts a method based on a biomechanical model.

The Hill muscle model is usually used as the biological model. Karavas et al. [110] proposed an auxiliary control based on remote impedance. Using sEMG signals and combining with the Hill muscle model, they directly calculated the required joint torque and joint stiffness trend, tracked the required stiffness and trajectory through the impedance controller, and studied stability under steady-state and transient conditions through experimental tests. At the same time, this model also allows the exoskeleton to generate auxiliary torque under the user’s command, improving the user’s autonomy. However, the current parameter calibration of the model is relatively complex, and only experiments on healthy subjects have been conducted. To accurately estimate the active torque of the ankle joint complex (AJC), Zhou et al. [111] proposed an online estimation framework based on the Hill musculoskeletal model and sEMG signals. By predicting muscle force through EMG signals and then converting it into joint torque, multi-DOF coupled active torque can be estimated online; combined with biomechanical and anatomical characteristics, the actively applied torque of multi-DOF coupled movements can be accurately estimated through a single-DOF identification–calibration strategy. Experimental results on five healthy subjects showed that this method had high estimation accuracy, with a single-DOF normalized root mean square error (NRMSE) of 10.29% and a multi-DOF NRMSE of 11.35%. However, to improve computational efficiency, the muscle model was simplified, focusing only on superficial muscles.

In addition to the Hill muscle model, Durandru et al. [112] estimated ankle torque in real time based on EMG signals and joint angles and a human neuromechanical model. A controller based on a disturbance observer can convert biological torque into exoskeleton commands. This method can achieve adaptive support for the user’s muscle activity under different gait conditions, significantly reducing biological joint torque (by approximately 22%) and EMG signal intensity (by 13%). Kiguchi et al. [113] correlated human joint movements with muscle activities and introduced a fuzzy neural modifier to adjust the components of the operation matrix to assist hip joint flexion/extension movements and knee joint flexion/extension movements. Experiments showed that the activity level of the tensor fasciae latae muscle was reduced with the help of the exoskeleton, which also proved the effectiveness of the controller.

In addition to model-based joint torque prediction, Meng et al. used EMG signals and joint angle signals to build a prediction model based on a TCN-LSTM hybrid neural network to predict the torque output of the knee joint exoskeleton robot in real time, introducing a joint attention mechanism to improve the accuracy of torque prediction, with an average mean absolute error (MAE) of 1.141 Nm. At the same time, the impedance control strategy was optimized to improve the effect of human–machine interaction. Although the coupling between muscles was not analyzed when building the model, it also provided a research direction for artificial intelligence-based torque prediction. In the future, the accuracy of the model can be further improved by analyzing the coupling between muscles to extract important features.

Although many studies have used EMG signals for real-time joint torque estimation, few studies have focused on how to improve the motion stability of EMG-based control methods and the performance of human–machine collaborative movements. Zhuang et al. [114] proposed an EMG-based Admittance Control Scheme (EACS), which improved stability through closed-loop PD control. Tests on twelve healthy participants and four stroke survivors in the ankle exoskeleton showed that it could stably and accurately track movements. Chen et al. [115] further optimized the admittance control, constructed an inner-outer double-loop admittance control, and proposed a new EMG-based two-layer admittance control. This method allows the subject to control the exoskeleton through the EMG signals of the thigh muscles while adapting to gait, and continuously and smoothly adjusts the gait trajectory through admittance control. In addition, an easy-to-use Myo thigh band and controller were proposed. The EMG signals of the thigh muscles were collected and applied to the hip and knee access control, simplifying the calibration process and significantly improving usability. Tests on six subjects showed that they could adapt to the target within an average of 16 steps, and could reduce muscle activation and human physical energy consumption.

Intention recognition and proportional myoelectric control based on sEMG signals have been put into practice. However, sEMG signals from different parts have different representation characteristics of lower limb joints. There are problems such as the complicated data combinations in the decoding process of multi-DOF joints and sEMG signals, which restrict the accuracy of exoskeleton robot control. Yuan et al. [116] proposed an interpretable multi-degree-of-freedom sEMG decoder based on additive mixed data enhancement. Based on the principle of linear superposition of sEMG signals in different degrees of freedom, the decoder generates synthetic multi-degree-of-freedom data by superimposing 1-DoF sEMG signals and force labels, using the deep forest model to perform decoding. Using only 1-DoF data can reduce regression errors by about 20%. This method has significant advantages in reducing data collection complexity and improving model generalization capabilities, but it still has shortcomings when dealing with degrees of freedom with special distribution or poor independence.

#### 3.4.2. Based on EEG Signals

EEG is a non-invasive technology for recording the electrical activity of neuronal populations in the brain, which is widely used in neurological disease monitoring and cognitive function research. Based on EEG acquisition, the BCI further integrates signal processing, feature extraction, and output control modules to build a closed-loop system for direct human–machine communication. EEG signals are obtained through dry or wet electrodes worn on the head. Their greatest advantage is that the data comes from the activity state between neurons in the patient’s brain, without requiring any feedback from the limbs, making them suitable for patients with severe motor disorders such as lower limb motor function loss and complete spinal cord injury.

Although BCI-based lower limb exoskeleton control has certain theoretical potential to bypass the damaged peripheral nervous system and directly drive movements from brain intentions, there are several challenges in practical applications: first, EEG signals have low spatial resolution, high noise, and are greatly affected by the environment and electrode stability, resulting in limited motion intention decoding accuracy; second, unlike EMG signals, there is a high delay from the generation to detection of EEG signals, which is not conducive to real-time control needs.

Although EMG-based motion intention decoding is subject to many limitations from hardware equipment and signal conditions themselves, it is still the main research and application path in the field of lower limb exoskeleton robot control. Vinoj et al. [117] designed a brain-controlled adaptive 6-DOF lower limb exoskeleton robot, which uses a non-invasive method to collect brain signals from the human scalp. A total of 16 electrodes are used for EEG sensors, and gold plating is used to improve conductivity. The collected signals are amplified and filtered for intention classification, and high-torque motors are driven to perform actions. The steady-state visual evoked potential (SSVEP) method was used to improve the classification accuracy (exceeding 80%), verifying the feasibility of controlling exoskeleton robots based on EEG signals, but the control accuracy and human–machine interaction effects were not discussed.

In the lower limb exoskeleton assistance system, pure EMG signals or pure EEG signals cannot adapt to the decoding of complex human movements, and the problem of mismatch between human–machine interaction control commands and actual actions restricts the effect of exoskeleton human–machine interaction. In response to this, Wang et al. [118] designed a multi-modal lower limb rehabilitation exoskeleton robot. EEG is used to obtain intention signals, and EMG is used to correct movement commands, realizing accurate recognition and correction of the patient’s movement intentions. A robust adaptive PD control system is used to improve the accuracy of action execution.

Targeting the problem of sEMG signals having the difficulty of distinguishing different movement amplitudes of the same movement, Li et al. [119] proposed a multi-modal human–machine interface design based on sEMG and EEG. By integrating the movement intention recognition of EEG and the action amplitude correction of sEMG, tests were conducted on 14 healthy subjects. The recognition accuracy of multi-modal fusion was 12% and 7.8% higher than that of using EEG or sEMG alone, respectively, with a maximum of 89.5%. However, for the exoskeleton robot, a simple state machine was used for control, which only included four actions and did not evaluate the action execution effect.

Overall, the performance of EEG-based exoskeleton control is relatively low, including usability, reliability, and decoding accuracy. Currently, it is mainly used as an auxiliary means to cooperate with EMG signals to achieve more accurate intention classification and intention recognition.

As the patient makes continuous progress in the rehabilitation process, the relevant control strategies will also be adjusted accordingly. In addition, since the perception, decision-making, and control of lower limb exoskeleton robots are an organic whole, the ability to use real and accurate human gait data further improves tracking accuracy and rehabilitation effects.

## 4. Discussion

Through the comparative analysis of control strategies in Section 3, different control strategies demonstrate varying advantages and limitations depending on the application scenario. Generally speaking, control strategies can be categorized as follows: passive control (PID, CTC), active control (impedance control, admittance control, sliding mode control), AAN control, and intelligent control (neural networks, fuzzy logic, etc.).

(a) Passive Control: It provides the highest kinematic accuracy and system stability (for example, Liang et al. [71] achieved an angle tracking error of <0.01 rad.), which is suitable for the early stages of rehabilitation where the exoskeleton assists in developing a standard gait pattern. However, it ignores the patient’s active participation, leading to reduced neuroplasticity; furthermore, the fixed trajectory may not perfectly match the user’s physiological structure, potentially causing discomfort.
(b) Active Tracking Control: It significantly improves human–machine interaction, enhancing comfort and adapting to individual gait differences. However, this method requires high-precision sensors and recognition algorithms, making it highly sensitive to model uncertainties and environmental disturbances (for example, Khamar et al. [84] reduced the impact of disturbances through BSC, resulting in a tracking error of <0.08 rad.).
(c) AAN (Adaptive Assistance and Navigation): It maximizes human participation. However, this strategy is highly dependent on parameter tuning, meaning that a set of parameters must be determined for each user.
(d) Intelligent Control: It achieves active control by predicting the user’s next movement, providing a more natural and intuitive user experience. However, it heavily relies on high-quality biological signals, making signal acquisition and decoding difficult.

Sometimes, although traditional passive control can achieve very low tracking errors, it cannot incorporate human intent into the control algorithm, thus failing to achieve optimal human–machine interaction. In contrast, combining control accuracy with human intent can lead to better human–machine collaboration. Currently, despite the wide range of applications offered by artificial intelligence algorithms, the lack of stability proofs and the high computational intensity remain major bottlenecks hindering their transition from simulation to real-world applications. This section provides a detailed review of the control strategies currently used in lower limb exoskeleton robots based on different control tasks. A summary of some control strategies is shown in Table 5.

When separated from specific control tasks, it can be found that there are similarities in the underlying end-control strategies, which can be specifically categorized into the following main types: PID control, sliding mode control, adaptive control, intelligent control algorithms, and hybrid control algorithms.

Overall, traditional position control methods (e.g., PID, SMC) are typically implemented by measuring motor angles via encoders and precisely adjusting controller outputs through closed-loop negative feedback control. They deliver favorable performance in the Human Motion Reproduction control task, enabling extremely high trajectory tracking accuracy. However, for the Human Motion Following control task, the high rigidity of hardware results in significant interactive resistance when human and robot gaits are inconsistent. As for the AAN control task, traditional control methods are difficult to achieve dynamic on-demand assistance due to their fixed parameters; only by combining traditional control with online adaptive control and intelligent control can on-demand assistance be realized.

The impedance control strategy is usually implemented based on torque feedback, which simulates the motor as a virtual spring–damper system. For the Human Motion Reproduction control task, it can also achieve trajectory tracking, but its accuracy is generally lower than that of PID control. For the Human Motion Following control task, real-time following is achieved by adjusting impedance parameters, which significantly reduces the interactive force. For the AAN control task, impedance control can also provide on-demand torque assistance by adjusting impedance gain parameters; yet, its performance is affected by motor inertia and mechanical structure, which requires continuous evaluation of parameter effectiveness.

Intelligent control strategies leverage neural networks to adjust motor current or gain in real time. They are capable of fulfilling all control tasks and feature strong individual adaptability. Nevertheless, such strategies demand substantial computing resources; delays caused by hardware can severely degrade human–machine interaction performance, and it is also difficult to rigorously prove their stability.

Currently, the unresolved common issues across the four types of tasks are as follows:

(a) Insufficient universality of control strategies;
(b) Lack of a standardized evaluation index system;
(c) Heavy reliance of advanced control methods on computing power/data.

Different control tasks place different emphases on elements such as perception, decision-making, control strategies, and structure. Currently, there are five main bottlenecks and constraints in the development of control strategies:

1. The control performance of exoskeleton robots not only depends on algorithm design, but is also closely related to the dynamic characteristics of the mechanical structure and drive system. Different control algorithms are difficult to use across platforms.

First of all, the driver type, layout, and transmission mechanism directly affect the damping, stiffness, and other structural parameters of the system, thereby changing the dynamic response characteristics and stability of the control system. This strong correlation between hardware and control results in significant differences in the performance of the same control strategy on different exoskeleton platforms, making it difficult to reuse across platforms.

To improve design flexibility, motors are usually placed directly on movable joints. However, the high number of degrees of freedom of leg movement joints leads to a substantial increase in the complexity of control strategies and costs. At the same time, motors also need to generate large auxiliary torques, which complicates the design of transmission mechanisms and reduction mechanisms. In recent years, many scholars have proposed compact design schemes for lower limb exoskeleton robots. Ishmael et al. [121] designed a four-bar linkage mechanism with an integrated composite spring for the motor-driven hip joint actuator of a lower limb exoskeleton. A compact carbon fiber frame surrounds the actuator and can also serve as the thigh link of the exoskeleton. A hierarchical controller was designed to achieve high-precision and high-output torque control. Performance test experiments showed that the device can generate high nominal torque (41.9 Nm repetitive peak torque), high backdrivability (0.16 Nm backdrive torque), high bandwidth (23.8 Hz), and high control precision (2.1% steady-state error). Experiments verified its application potential in daily walking and stair assistance.

In addition to hardware innovation, improving dynamic control performance is also critical. Currently, most experiments focus on optimizing motor control accuracy and torque disturbance suppression, while there is relatively little research on dynamic performance and transition processes. The dynamic performance of the motor’s output torque in the lower limb exoskeleton system directly affects the effect of human–machine interaction. Wu et al. [122] focused on the dynamic execution process of motor-driven exoskeleton robots and designed an improved reaching law sliding mode controller with high control precision and good control quality, which improved the smoothness and response speed of the lower limb exoskeleton during dynamic processes and made human–machine interaction more compliant.

In addition to using algorithms to optimize the control parameters of exoskeleton robots as mentioned above, some scholars have proposed algorithms to optimize the mechanical structure design of exoskeletons in recent years. Genetic algorithms can be used to optimize the mechanical design parameters of exoskeletons. For the case of only one objective function, Du et al. [123] used the GA to optimize the link length, and Tschiersky et al. [124] used the GA to find the best actuator placement position to improve the driving efficiency. For the case of multiple objective functions existing at the same time and needing to be optimized at the same time, the improved GA can also design the optimal mechanical structure parameters. The design optimization of exoskeleton robots covers multiple dimensions such as control strategy, mechanical structure, and parameter configuration. Its core is to achieve multi-objective balance through the optimal combination of design variables, improve output torque, minimize system weight, and improve human–machine adaptation comfort [125]. Traditional optimization methods have obvious limitations in dealing with multi-objective constraints and strong nonlinear characteristics, and it is difficult to break through the dilemma of local optimal solutions. Evolutionary computation (EC) has shown unique advantages in the field of global optimization due to its swarm intelligence characteristics. By combining it with the policy search capabilities of deep learning, it is expected to build a multi-objective collaborative optimization framework, which will promote the systematic optimization of exoskeleton robots in terms of motion performance, structural efficiency, and human–computer interaction.

With the application of methods such as digital twins and reinforcement learning in the industrial field, more and more digital models are being integrated into the information interaction between users and equipment. This allows the control performance of exoskeleton robots to overcome the limitations of the physical environment. Luo et al. [126] achieved this by learning in a purely simulated environment, abandoning traditional data acquisition and curve fitting methods, and integrating the simulation results into a physical model. This reduces reliance on human experiments; by introducing reinforcement learning, it improves control accuracy and training efficiency. Beyond modeling, digital twin technology can also act as a “virtual sensor,” using high-precision simulation models to reduce reliance on physical sensors.

2. A single driving method cannot meet the requirements of complex scenarios.

With the diversification of exoskeleton robot requirements, a single drive mode has encountered bottlenecks in terms of compliance and weight. Different drive modes have their own advantages and limitations. Integrating multiple drive modes to form a hybrid power drive system is expected to bring new breakthroughs to the control of lower limb exoskeleton robots.

Luo et al. [127] introduced pneumatic muscles to provide gravity support on the basis of traditional mechanical support, forming an electro-pneumatic hybrid drive mode, which can not only control torque accurately but also reduce the weight of the exoskeleton itself. Simulation results show that the control error of each joint is less than ±1.5°. Sun et al. [128] used shape memory alloy (SMA) and motors to design a knee–ankle orthosis for lower limb rehabilitation. SMA can provide driving force and reverse damping force without an electrical structure, with a large power density ratio and small volume. It can also be used in flexible wearable exoskeleton design to achieve a rigid–flexible coupled structure. The output of the SMA actuator was evaluated through experiments, and the results were consistent with the theoretical model. Subsequently, the research team proposed an estimation method for determining joint torque for this structure [129], and conducted prototype and human experiments; the torque estimation error during knee joint movement was reduced by 59.1%, 16.5%, and 73%, which can meet the requirements of daily movement assistance for the lower limbs.

Currently, there are relatively few studies on the hybrid drive design of lower limb exoskeleton robots. Wang et al. [130] designed a hybrid multi-degree-of-freedom upper limb exoskeleton rehabilitation training device mainly driven by pneumatic muscles and supplemented by motor drive. Joints with a large range of motion still use motor drive, while the flexion and extension of the shoulder and elbow joints and the rotation of the wrist joint are driven by pneumatic muscles, which balances portability and safety, providing new ideas and research directions for the hybrid drive of lower limb exoskeleton robots. At the same time, developing lightweight pneumatic or low-power high-torque motor drives is the key to reducing costs and one of the focuses of the commercialization of lower limb exoskeleton robots in the future.

Another type of hybrid drive mainly combines functional electrical stimulation with exoskeleton robots. Del-Ama et al. [131] proposed a hybrid FES-robot collaborative control strategy, which uses iterative learning control (ILC) and closed-loop PID control to adjust the electrical stimulation parameters of the knee flexors and extensors, balance the power output of the exoskeleton and muscles, and minimize the interaction torque between the exoskeleton and the user. It was applied to the Kinesis, a single-degree-of-freedom knee exoskeleton robot, and effectively reduced the assistance of the exoskeleton while ensuring accurate motion trajectories. This method is suitable for the gait rehabilitation of patients with low-level spinal cord injuries. However, currently, only healthy subjects have been tested, and there is a gap in its application to patients with spinal cord injuries.

3. Insufficient Universality and Adaptability of Control Strategy Design, with Obvious Innovation Bottlenecks at the Algorithm Level.

When faced with patients with complex terrain, variable speed, or different rehabilitation processes, the controller often cannot automatically adjust the parameters, resulting in a decrease in performance. Online adaptive frameworks can address this problem to some extent. Kang et al. [132] used real-time data streams to continuously update user state estimation models, effectively customizing models for new users by learning user-specific gait patterns. Different models can match both healthy individuals and patients, showing high potential for personalized assistance in clinical applications. However, this method is still limited by the models themselves. The control strategy based on AI algorithm is data-driven and can reduce dependency on the model, which is the “general control strategy”. Lee et al. [133] proposed an AI-driven general lower limb exoskeleton control system, which can capture external and internal states for real-time control, adjust the auxiliary torque according to the user and environmental state, and adapt to the user’s preferences. The experimental results show that compared with the traditional auxiliary method, this method reduces the human metabolic cost by 6.5%, but there is no detailed and in-depth study on the accuracy of gait adjustment. Emerging methods such as deep learning and reinforcement learning can learn control strategies from big data, dynamically adjusting the human–robot interaction force to ensure that the exoskeleton remains compliant with the body’s natural gait during movement. Rose et al. [134] first proposed an end-to-end model-free deep reinforcement learning method that can not only learn to follow desired gait patterns but also consider the user’s existing gait patterns, exhibiting strong disturbance rejection and high robustness, this method has proven effective in the personalization of gait training. At the same time, the calculation process of AI general control algorithm is computationally intensive, and the global stability is difficult to rigorously prove. This makes the interpretability and verifiability of the algorithm an urgent problem to be solved. In addition, there is a gap between the simulation of the general control algorithm and the actual control effect, and theoretical development far exceeds its application in the control layer. Therefore, improving the adaptability and generalization performance of control strategies, combined with real-time perception and intelligent adjustment mechanisms, has become the focus and challenge of the current research. This will not only help enhance the adaptability of exoskeleton systems to diverse usage environments, but also improve user experience and rehabilitation outcomes.

4. Limitations in Testing Scenarios and Inadequate Attention to Human–Machine Interaction Evaluation.

Currently, testing for exoskeleton robots mainly focuses on flat-ground walking and treadmill walking, providing leg assistance to users [135]. Upright walking is one of the main functional requirements for lower limb exoskeletons, but this does not mean that upright walking has low relevance to other lower limb movements—such as running, squatting, jumping, and leg shaking. To cover these movements, exoskeleton control strategies must achieve precise execution of actions in real environments, and must consider balance performance on special terrains (e.g., grass, sand, slopes). This places higher demands on the self-learning and adaptability of lower limb exoskeleton control strategies. Traditional model-based controllers often struggle to cope with the uncertainties of uneven surfaces. Guo et al. [136] used the Twin Delayed Deep Deterministic Policy Gradient (TD3) algorithm to achieve gait training control for an exoskeleton during stair climbing, but this control was limited to the sagittal plane. Guo et al. [137] from the University of Electronic Science and Technology of China used neural network regression to identify slope parameters in urban multi-terrain environments, relying solely on IMUs, providing the exoskeleton with real-time terrain awareness and adaptive assistance during stair climbing and while walking on steep slopes.

In addition to the objective performance description of lower limb exoskeleton robots, the evaluation of human–machine interaction effects is also a key test indicator for exoskeleton robot evaluation. Whether the control effect meets the usage habits of different users and whether compliant control can be achieved should also receive high attention.

## 5. Conclusions

This paper provides a systematic review of control strategies for the rehabilitation of lower limb exoskeleton robots. Based on a hierarchical control architecture, it comprehensively outlines the technical approaches and implementation mechanisms of core components of exoskeleton robots. The paper begins by summarizing the applications of different drive methods in rehabilitation exoskeletons, focusing on the widely used motor drive method and analyzing the structural characteristics of related control systems.

Subsequently, combined with four types of control tasks—“motion reproduction”, “motion following”, “AAN”, and “intention perception”—it systematically concludes the basic principles, application characteristics, and respective advantages and limitations of different strategies. Based on this, we further extracted and summarized the common technical elements and main constraints in the current exoskeleton control strategy from aspects such as motor installation and system coupling, multi-source sensing and human–machine design, adaptability and universality of control methods, and physical constraints of algorithms.

In summary, the control task has evolved from “open-loop trajectory → human–machine collaboration → intent prediction,” and the driving strategy has transitioned from PID control to adaptive/AI fusion. However, the generalization ability of the control strategy remains a core challenge. This paper aims to provide a structured overview and practical reference for the research of control strategies for lower limb exoskeleton robots, offering theoretical support and technical basis for subsequent control strategy design, performance evaluation, and system integration. Although breakthroughs have been achieved in many aspects of control strategies, challenges such as high cost, technical limitations, and safety issues still exist. Therefore, continuous research and innovation in control strategy optimization are necessary. Solving these problems is crucial to ensuring that advanced control strategies can significantly improve the function of lower limb exoskeletons.

## Figures and Tables

**Table 1:** Partial table of lower limb exoskeleton robot products.

Research Team/Researcher	Model	Joints Involved	Application Scenario	Drive Method	Technical Features/Advantages
U.S. Lockheed Martin (Washington, DC, USA)	Onyx Exoskeleton	Feet, knees and hips	Tasks requiring high physical effort, involving repeated kneeling, squatting, climbing, and lifting heavy objects.	Motor	Fast response speed (150 ms) and high load-bearing capacity, assisting users in performing 72 squats while carrying a load of 185 pounds.
U.S. Dephy (Boxborough, MA, USA)	Exo-boot	Foot, Ankle	Military-powered exoskeleton.	Inflatable actuator	It provides ankle exoskeleton torque prior to the body’s natural reaction, enabling subjects to withstand 9% greater external disturbances.
RexBionic (Auckland, New Zealand)	Rex	Hip, Knee, Ankle	Squat, lunge, sit-to-stand test, leg swing, and stretching exercise.	Motor	Equipped with 27 on-board microprocessors to ensure stability during movement; it is suitable for people with a large body weight (100 kg).
Panasonic (Osaka, Japan)	ATOUN MODEL Y	Lumbar Region	Workplaces that can reduce waist strain and are used for lifting and lowering heavy objects.	Motor	Lightweight (10 pounds) and meeting waterproof and dustproof requirements.
University of Tsukuba (Tokyo, Japan)	HAL-5	Hip, Knee, Ankle	Standing, walking, climbing, grasping, lifting heavy objects, etc.	Motor	The world’s first safety-certified exoskeleton.
Beijing AI- robotics Technology Co., Ltd (Beijing, China)	AiWalker	Hip, Knee, Ankle	Full-series rehabilitative exoskeleton robots, suitable for the entire cycle of rehabilitation process for patients with spinal cord injuries.	Motor	It perfectly adapts to patients in different rehabilitation stages and supports multiple scenarios and body types.
Shanghai ULSrobotics (Shanghai, China)	BES-P	Hip, Knee	Alternative lifting equipment.	Energy storage drive	Lightweight (3.6 kg) and compact in size, it imposes no extra burden on the body.
	FIT-GS-PRO	Hip, Knee, Ankle	Human augmentation, assisted walking, and medical research and development.	Servo motor	Featuring multiple functions to meet the needs of different scenarios and a high degree of freedom; it supports secondary development.
Shanghai Siyi Intelligence Technology Co., Ltd. (Shanghai, China)	EasyWalk^®^X1	Ankle	Used to address walking disorders in patients with stroke, traumatic brain injury, etc., and provide assistance to help patients with ankle dorsiflexion and plantarflexion.	Cable drive	Flexible actuation that does not restrict the human body’s free movement.
Shanghai Fourier (Shanghai, China)	ExoMotus	Hip, Knee	Auxiliary support	Motor	Integrated ergonomic design with customizable parameters to meet the requirements of different rehabilitation training programs.
Shenzhen Zuowei Technology Co., Ltd. (Shenzhen, China)	Intelligent Walking-Assistance Robot	Hip	Walking assistance mode, hemiplegia mode, Parkinson’s mode. These are used to assist stroke patients in their daily rehabilitation training.	Brushless dc motor	It has a proprietary assessment function, adapts to different populations, and is easy to wear—donning and doffing can be completed in 30 s without assistance.
Harvard University (Cambridge, MA, USA)	Soft Exosuits	Hip, Knee	Sitting, standing, walking, going up/down slopes, going up/down stairs, running, jumping.	Cable drive	The wearer’s joints are not restricted by external rigid structures, and the wearable component is extremely lightweight.
Nanjing Institute of Technology (Nanjing, China)	Prototype	Hip, Knee	Walking assistance.	Hydraulic drive	Hydraulic rods are adopted at the joints to drive the connecting rods of the thighs and calves, which boasts the advantages of stable operation and overload protection.

**Table 2:** Comparison of driving methods.

Category	Feature	Advantage	Limitation	Application Scenario	Response Speed	References
Motor	High precision, low latency, and closed-loop controllable; it can achieve a high-torque drive when matched with a reducer.	Compact in size, simple to maintain, and low in power consumption; suitable for high-precision motion control.	High-precision motors are expensive; complex control algorithms are required for high-precision demands.	Rehabilitation exoskeleton, daily assistive exoskeleton, precise joint control	Fast	[38,39,40,41]
Hydraulics	High power density and large driving force; it usually requires hydraulic pumps, valves, pipelines, and fluid storage tanks.	Suitable for high-intensity industrial applications; stable output force and strong impact resistance.	Complex system, heavy weight, high maintenance cost; high power consumption.	Industrial/military exoskeletons, static scenarios requiring high torque	Medium	[42,43,44,45]
Cylinder	Linear drive, dependent on compressed gas.	Low cost, simple structure, no pollution; suitable for short-term high-explosive-force scenarios.	Limited compressed gas storage; bulky and noisy compressors; delays caused by gas compressibility.	Assisting sitting–standing movements, short-term rehabilitation training, soft exoskeletons	Slow	[46,47]
Pneumatic Artificial Muscles (PAMs)	Flexible drive, which generates pulling force through pneumatic contraction.	Lightweight, high compliance, and good bionic performance; suitable for synergy with the body’s natural movements.	Only capable of one-way drive (requires paired use), with complex pneumatic control; dependent on high power compressors.	Soft exoskeletons	Slow	[48,49,50,51]
Cable-driven	Power is transmitted via cables, allowing actuators to be placed away from joints.	Lightweight; suitable for multi-degree-of-freedom coordinated control.	High friction loss in cables, which are prone to wear after long-term use; requires matching with motors or pneumatic actuators; usually only capable of pulling rather than extending.	Lightweight exoskeletons, gait training equipment	Medium	[52,53,54]
Elastic Actuators	Passive (e.g., springs) or semi-active (e.g., shape memory alloys); energy storage and release.	Zero power consumption, low cost, and high reliability; can absorb shocks and reduce motor load.	Cannot actively adjust the output force; poor adaptability when used alone and needs to be combined with other actuators.	Passive assistive exoskeletons	Fast	[55,56]

**Table 3:** Comparison of control tasks.

Control Task	Control Objective	Typical Control Strategy	Required Sensors	Typical Application Phase
Reproduction of human movement	Accurate reproduction of preset trajectories	Trajectory tracking control, position control, open-loop force control	Motor encoder, Inertial Measurement Unit (IMU)	Early-stage rehabilitation, passive training
Following human movement	Real-time synchronization with human movements	Interactive force feedback control, EMG-driven, impedance control	Force sensor, EMG	Mid-stage rehabilitation, users with active ability
Joint Torque Assist-As-Needed	Provision of timely assistance	Inverse dynamics model, fitted torque assistance, finite state control	Joint angle sensor, EMG sensor	Late-stage rehabilitation, movement assistance
Motion prediction and compliant control	Intelligent intent recognition + compliant control	AI predictive control, fuzzy logic, adaptive impedance	Electroencephalography (EEG) sensor, EMG sensor, brain–computer interface (BCI), force sensor, angle sensor	Advanced-stage rehabilitation, daily assist exoskeleton

**Table 4:** Comparison of motion trajectory acquisition methods.

Method Category	Basic Principle	Advantages	Disadvantages	References
Predefined Trajectory Method	Utilize clinical medicine databases or collect healthy human movement curves as reference trajectories	Simple and easy to use, suitable for standardized rehabilitation training	Lacks individualization and cannot adapt to patient differences or sudden disturbances.	[58,59,60,61]
Mathematical Model Method	Derive trajectories based on simplified human body models (such as inverted pendulum models, Zero-Moment Point models)	Clear model structure, suitable for systematic analysis	Accuracy is limited by model simplification assumptions, making it difficult to handle complex gaits or actual human differences.	[62,63,64]
Gait Planning Method	Draw on industrial robot trajectory planning theories to optimize trajectories under multi-objective and constraint conditions	Smooth trajectory, suitable for high-degree-of-freedom systems	Complex computation, poor real-time performance, and weak adaptability to dynamic changes.	[65,66]
Machine Learning-Based Prediction Method	Use neural networks or regression models to learn users’ historical gait data and predict future trajectories	Enables individualized and dynamic prediction with strong adaptability	High dependence on data and limited generalization ability.	[67,68,69,70]

**Table 5:** Comparison of control strategies.

Control Strategy/Innovation	References	DOF	Advantages	Applicable Scenarios	Limitations
**Human Motion Following**					
Exoskeleton PD Closed-Loop Control	[85]	2	High control accuracy	Verifies the effectiveness of PD control in low-complexity exoskeleton systems	Simulation-only
Virtual Model Control Algorithm (VMC)	[87]	/	High computational efficiency, significantly reduced human–machine interaction force, and improved wearing comfort	Heavy-load assistive lower limb exoskeleton, especially suitable for continuous gait control and dynamic collaboration	Difficulty handling abrupt dynamics; simulation testing only
Adaptive Robust Control Based on Udwadia–Kalaba Theory	[86]	2	No linearization or approximate approximation required	Applicable to exoskeleton tracking control scenarios with multiple time-varying disturbances	Complex parameter tuning and lack of adaptability. Simulation-only
Feedforward Neural Network	[88]	3	Only position information needed, capable of compensating for unknown nonlinear dynamics of the system and improving control accuracy	Suitable for rehabilitation training and gait assistance of lower limb exoskeleton robots, especially in scenarios that require precise trajectory tracking but lack speed sensors	The gain settings were highly variable and only single-subject experiments were conducted; no clinical trials were performed
Neural Network + Repetitive Learning	[89]	2	Improved tracking accuracy, enhanced transient performance, and effective handling of periodic and aperiodic uncertainties	Suitable for rehabilitation training, demanding high precision and fast response	Simulation-only
FCMAC + CTC	[90]	3	Capable of achieving accurate trajectory tracking even when model parameters and loads change, with high tolerance for uncertainties	Applicable to scenarios with frequent load changes and external disturbances	When the system uncertainty is too large, the proposed control method cannot guarantee tracking performance; simulation-only
RBF-FVI	[91]	6	Combination of fuzzy PID control module and RBF neural network compensation module significantly improves system compliance and trajectory tracking accuracy	Minimizes exoskeleton assistance to enhance patient participation	Torque mutation issue exists during gait phase transition, and testing is only conducted on healthy users
Fuzzy Adaptive Sliding Mode Control	[92]	2	Suppresses chattering, improves tracking accuracy, features fast response speed, and strong robustness	Suitable for human–machine collaboration systems with significant dynamic uncertainties and high-precision trajectory tracking requirements	Fuzzy rule design relies on experience, with a complex system structure
**Joint Torque Assist-As-Needed**					
Deep Reinforcement Learning	[120]	8	The controller can adapt to the muscle conditions of different patients	Suitable for scenarios requiring adaptation to different patients’ muscle conditions and neuromuscular diseases	Simulation-only
Spring + Intelligent Clutch	[97,98]	1	The adaptive clutch enables precise power assistance	Flat-ground walking and short-distance load-carrying tasks	Structural deviation is prone to occur; the spring stiffness range and material need optimization; testing was only conducted on healthy subjects
Optimization of Energy Storage Components	[101]	/	Specific walking assistance can be provided to patients by adjusting the energy storage components. The test was conducted on a patient with complete spinal cord injury	Power assistance for patients with spinal cord injuries	It assumes that the exoskeleton’s human-attached modeling and joint kinematics remain unchanged, with a slight increase in the activation level of lower back muscles
Muscle Force Synergy Compensation Strategy	[103]	2	Joint assistance is delivered along the muscle force compensation path to maximize the utilization efficiency of gait energy	Reduces metabolic energy consumption during human walking	There is a contradiction between the linear spring and the human body’s nonlinear movement; vibration occurs during spring energy release. Single-person experiments were conducted
Adopting PAMs (Pneumatic Artificial Muscles) to Simulate the Nonlinear Characteristics of Human Tendons	[99]	1	It features lightweight design, and its nonlinear components are more consistent with human body characteristics	Applicable to users with different gaits	The matching degree between PAM stiffness and the user needs further optimization; kinematic modeling is only simulated based on the gait data of healthy individuals
**Perception–Prediction of Human Motion and Compliant Control**					
Proportional EMG fixed gain control	[105]	2	Control is smooth, which can significantly reduce muscle activity and metabolic consumption	This experiment mainly verifies the feasibility of proportional EMG control. The test was conducted on 10 healthy subjects	Verification was only conducted on healthy subjects during flat-ground walking
Proportional EMG control	[106]	1	High trajectory tracking accuracy	Effectively controls knee joint movement during non-weight-bearing activities. The approach was implemented on three transfemoral amputees	Testing was only performed in a sitting posture
Surface EMG signal-based gain adjustment compliance control strategy	[108]	/	Significantly enhances the compliance of the robot end-effector and improves the safety of the robot in different training modes	Improves the compliance of the training process	Normalized thresholds require manual setting, and the training mode is relatively single. The testing was conducted by a single person and only on the prototype device
Hip exoskeleton gastrocnemius EMG control	[109]	1	Improves the synchronization of assistance and user comfort, and reduces motion displacement difference	Gait adjustment during walking	Controller parameters are adjusted based on the user’s subjective perception, without considering reliability for all users
the Hill-based sEMG-force model	[111]	/	High prediction accuracy and good real-time performance	Provides accurate actively applied torque data, thereby contributing to early disease diagnosis and patient-specific treatment plans	No focus on deep muscles leads to information loss, making it impossible to fully capture inter-subject differences in musculoskeletal parameters
Neuromechanical model-based control (NMBC)	[112]	1	No predefined torque trajectory or state machine is required, and it adapts to various gait conditions	Scenarios with complex gait changes and high adaptability requirements. The test was conducted on six healthy subjects	Testing was only conducted on healthy subjects
EMG-based admittance control	[114]	1	Dynamically adjusts the reference trajectory and compensates for unmodeled factors through a closed-loop structure to improve stability	Rehabilitation of patients with hemiplegia. The test was conducted on twelve healthy subjects and four patients with ankle injuries	Relies on individual calibration and EMG signal quality
Novel two-layer admittance control based on EMG	[115]	4	Allows subjects to continuously adjust the gait trajectory, with better adaptation range and adaptation time of gait trajectory parameters	Walking rehabilitation therapy. The test was conducted on six healthy subjects	Only data from a single leg was collected, and all data were obtained from healthy subjects
Lower limb exoskeleton controlled by EEG signals	[117]	6	High classification accuracy and ability to identify the movement state of paralyzed patients	Patients with severe paralysis	Control accuracy and human–machine interaction effects were not discussed. The test was conducted on only one healthy subject
Multi-modal robust adaptive PD control	[118]	2	The combination of EEG and EMG signals makes intention recognition more accurate	Remodeling of the motor nervous system in stroke patients	Testing was only performed on squatting movements. The test was conducted on only one healthy subject

## Data Availability

No new data were created or analyzed in this study.
